# Compound kushen injection suppresses human acute myeloid leukaemia by regulating the Prdxs/ROS/Trx1 signalling pathway

**DOI:** 10.1186/s13046-018-0948-3

**Published:** 2018-11-19

**Authors:** Yanxia Jin, Qian Yang, Li Liang, Lu Ding, Yuxing Liang, Dongdong Zhang, Balu Wu, Tian Yang, Hailing Liu, Tingting Huang, Hui Shen, Honglei Tu, Yunbao Pan, Yongchang Wei, Yi Yang, Fuling Zhou

**Affiliations:** 1 Department of Haematology, Zhongnan Hospital, Wuhan University, No. 169 Donghu Road, Wuchang District, Wuhan, 430071 Hubei Province China; 2 Key Laboratory of Artificial Micro- and Nano-Structures of Ministry of Education, School of Physics and Technology, Wuhan University, Wuhan, 430072 Hubei China; 3 Department of Radiation and Medical Oncology, Zhongnan Hospital of Wuhan University, Wuhan, 430071 Hubei China; 4 Department of Clinical Haematology, Second Affiliated Hospital, Medical School of Xi’an Jiaotong University, Xi’an, 710004 Shaanxi China; 5 Department of Laboratory Medicine, Zhongnan Hospital, Wuhan University, Wuhan, 430071 Hubei China

**Keywords:** Acute myeloid leukaemia, Reactive oxygen species, Compound kushen injection, Peroxiredoxin-3, Peroxiredoxin-2

## Abstract

**Background:**

The increase in the levels of reactive oxygen species (ROS) in acute myeloid leukemia (AML) patients has been previously described; thus, it is important to regulate ROS levels in AML.

**Methods:**

Flow cytometry were used to assess the in vitro effect of compound kushen injection (CKI). Quantitative proteomics were used to analyse the mechanism. The AML patient-derived xenograft (PDX) model were used to evaluate the in vivo effect of CKI.

**Results:**

We found that intracellular ROS levels in AML cells were decreased, the antioxidant capacity were increased when treated with CKI. CKI inhibited the proliferation of AML cells and enhanced the cytotoxicity of AML cells, which has few toxic effects on haematopoietic stem cells (HSCs) and T cells. At the single-cell level, individual AML cells died gradually by CKI treatment on optofluidic chips. CKI promoted apoptosis and arrested cell cycle at G1/G0 phase in U937 cells. Furthermore, higher peroxiredoxin-3 (Prdx3) expression levels were identified in CKI-treated U937 cells through quantitative proteomics detection. Mechanically, the expression of Prdx3 and peroxiredoxin-2 (Prdx2) was up-regulated in CKI-treated AML cells, while thioredoxin 1 (Trx1) was reduced. Laser confocal microscopy showed that the proteins Prdx2 could be Interacted with Trx1 by CKI treatment. In vivo, the survival was longer and the disease was partially alleviated by decreased CD45+ immunophenotyping in peripheral blood in the CKI-treated group in the AML PDX model.

**Conclusions:**

Antioxidant CKI possess better clinical application against AML through the Prdxs/ROS/Trx1 signalling pathway.

**Electronic supplementary material:**

The online version of this article (10.1186/s13046-018-0948-3) contains supplementary material, which is available to authorized users.

## Background

Acute myeloid leukaemia (AML) is a haematological malignant hyperplastic disease that originates in haematopoietic stem cells. A large number of abnormal cells in bone marrow accumulate and infiltrate other tissues due to out-of-control proliferation and differentiation, resulting in the inhibition of normal haematopoietic function and poor prognosis in AML patients. Inducing chemotherapy to inhibit AML cell proliferation and further strengthening chemotherapy to keep AML patients in a remission state is the main method for treating AML in the clinic. However, chemotherapy can lead to bone marrow failure in AML patients because of the many toxic side effects and chemotherapy complications, even due to bleeding or severe infection resulting in death, which are difficult challenges for the clinical treatment of AML.

Reactive oxygen species (ROS) are a second messenger that regulate normal physiological processes and maintain the intracellular REDOX balance. An ROS imbalance causes hydroxyl radicals to attack the DNA skeleton, causing DNA oxidation damage and increasing DNA mutation, leading to genome instability and cancer [1]. Hole et al. proposed that more than 60% of AML patients had sustained NADPH oxidase (NOX) activation, leading to high ROS accumulation and increased AML cell proliferation [2]. An increase in ROS levels is closely related to DNA oxidative damage and mutation in the development of AML [3]. In our previous study, we found that ROS is involved in the development of AML [4]. ROS levels were significantly increased in AML patients, the total antioxidant capacity (T-AOC) was decreased and the levels of the oxidative damage index 8-hydroxydeoxyguanosine (8-oH-dG) were increased, which accelerated the progression of AML [5]. Similar to our previous reports, the increased ROS levels in AML patient relapse induced higher promotor oncogene c-Jun activation domain-binding protein l (*Jab1*) and thioredoxin 1 (*Trx1)* expression; moreover, *Jab1* binding to *Trx1* affected Trx1 [6]. The higher ROS levels and Jab1 and Trx1 expression were significantly positively correlated with poor survival in AML patients, which promoted malignant proliferation in AML cells. Therefore, regulating the ROS signalling pathway will be a promising strategy for AML treatment.

**Fig. 1:**
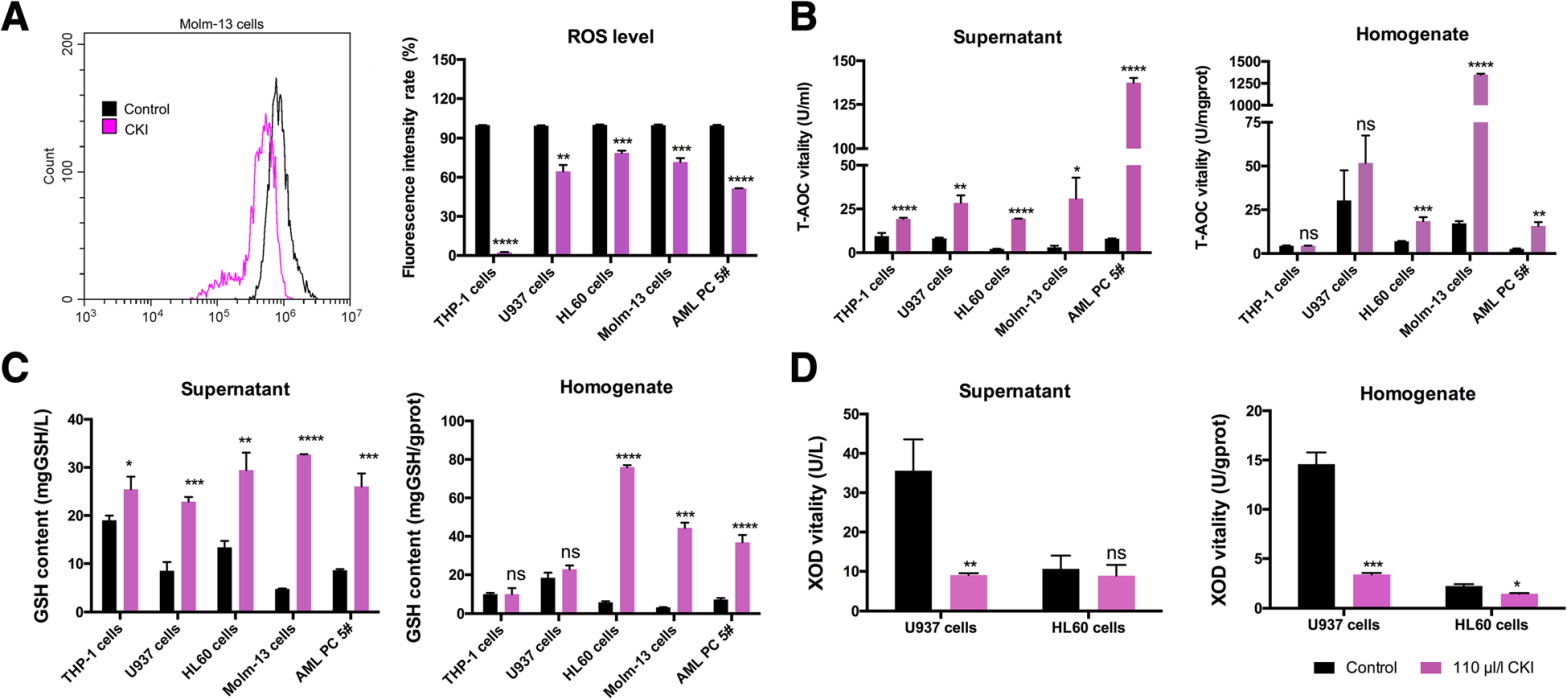
The antioxidant CKI decreased intracellular ROS levels in AML cells. **a** Statistical analysis of the intracellular ROS levels after AML cells were treated with 110 μl/ml CKI for 24 h. **b**, **c**, **d** The ROS-related indexes were tested using AML cell supernatants and homogenates with kits as described in the methods. **b** T-AOC vitality. **c** GSH contents. **d** XOD vitality

**Fig. 2:**
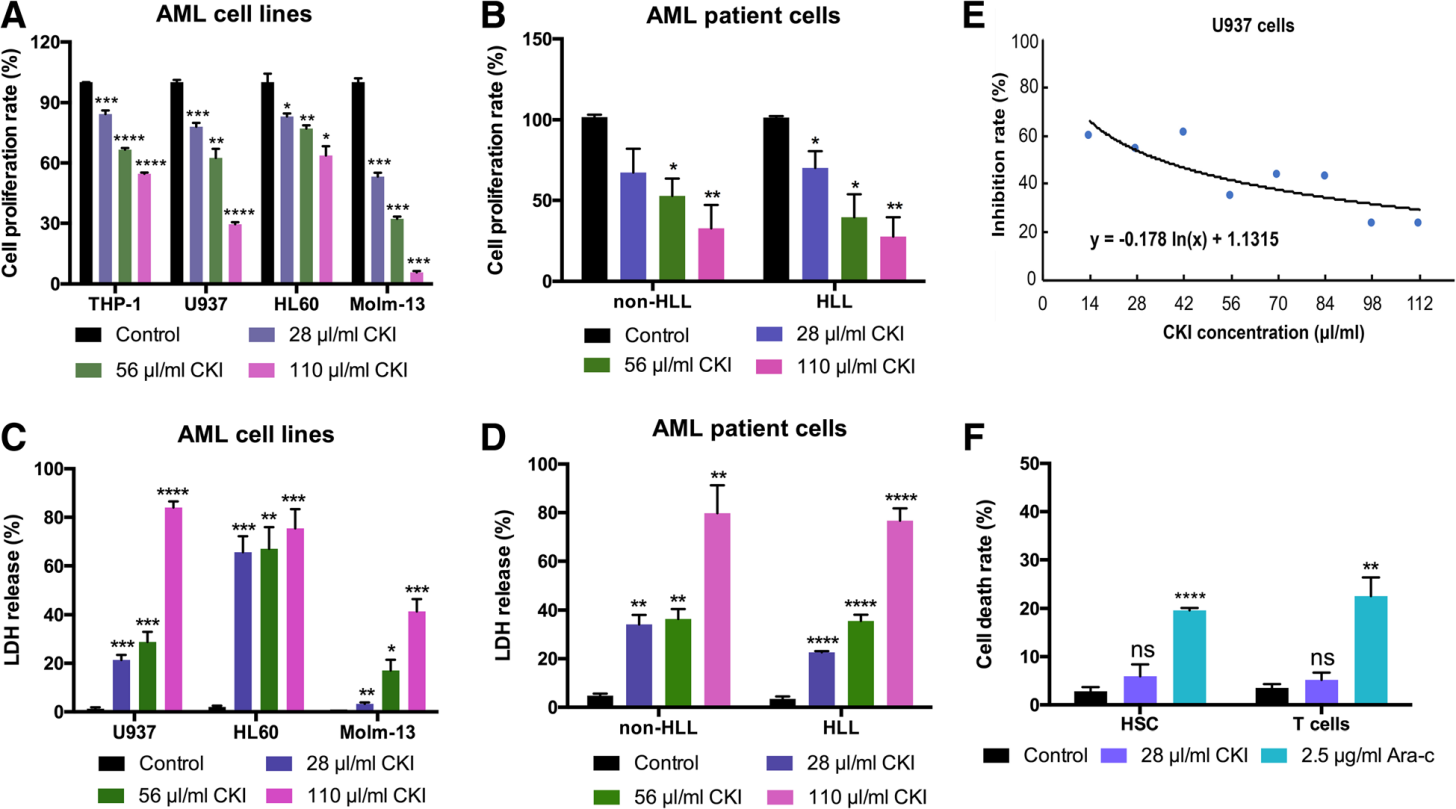
The compound kushen injection inhibited AML cell proliferation. **a**, **b** Test the cell proliferation of four AML cells (**a**) or (**b**) human patient cells isolated from AML patients were treated with three doses of CKI at 28 μl/ml, 56 μl/ml, and 110 μl/ml for 48 h using CCK8 kits. **c**, **d** Test the cytotoxicity and cell pore-forming activity of three AML cell lines (**c**) and human patient cells (**d**) were treated with three doses of CKI at 28 μl/ml, 56 μl/ml, and 110 μl/ml for 48 h by LDH release assay. **e** The inhibition effects of CKI were examined in U937 cells at different doses for 48 h, and the half maximal inhibitory concentration (IC50) values was determined. **f** The cell death rate was tested by MTT assay. Haematopoietic stem cells (HSCs) and T cells were treated with 28 μl/ml CKI or 2.5 mg/ml Ara-c for 48 h. HLL, hyperleukocytic leukaemia. Bars represent ±SEM

In view of these results, we believe that reducing ROS levels and regulating the ROS pathway with high efficiency and low toxicity antioxidants will effectively improve survival in AML patients [7]. We have explored and screened a series of antioxidants and found that the compound kushen injection (CKI) could decrease intracellular ROS levels and inhibit AML cell proliferation (Figs. 1 and 2). CKI is a compound containing oxymatrine and matrine, which are mainly used to stop in cancer pain and bleeding and had no obvious toxic side effects. The component of CKI is shown in Additional file 1: Figure S1. Studies have shown that CKI has antioxidant and immune activities and inhibits the occurrence of gastric cancer by enhancing immune activity [8]. In vitro and in vivo studies have confirmed that CKI inhibits human breast cancer stem cell proliferation by down-regulating the Wnt/β-catenin pathway [9]. Additionally, CKI reduces tumour growth and alleviates cancer pain by blocking the TRPVI signalling pathway [10].

In the clinic, our team has reported that CKI combined chemotherapy could effectively treat acute leukaemia, and 81.8% of AML patients could achieve complete remission; importantly, CKI could decrease the toxic side effects associated with chemotherapy [11]. However, the CKI molecular mechanism against AML remains unclear. In this study, we analysed the treatment efficacy and potential mechanism of CKI on AML in vitro and in vivo by observing the changes in single-cell morphology using single-cell optofluidics technology, validated the changes in ROS-related targets using Western blot and laser confocal microscopy, and evaluated the treatment effects on AML mice through construction of an AML animal model and AML patient-derived xenograft model. We aimed to explore the therapeutic efficacy of the low-toxic natural antioxidant CKI against AML by regulating ROS pathways and providing a new strategy for enhancing AML patient survival.

## Methods

### Cell culture and sample collection

Acute myeloid leukaemia THP-1, U937, HL60 and Molm-13 cells were obtained from the cell resource centre from Shanghai Institutes for Biological Sciences (Shanghai, China) and maintained in PRMI 1640 media containing 10% foetal bovine serum (Gibco, Invitrogen, Carlsbad, CA, USA) with 100 U/ml penicillin and 100 μg/ml streptomycin at 37 °C and 5% CO_2_.

Human cells were collected from 36 AML patients and 15 healthy controls; information on the collected clinical samples is provided in Additional file 2: Table S1. CD34+ cells were separated with anti-CD34+ antibody-coated magnetic microbeads (MACS, Miltenyi Biotec, Germany). Patient diagnosis with AML was based on standard morphological and cytochemical examinations of peripheral blood and marrow smears according to the French-American-British (FAB) and World Health Organization (WHO) criteria [12–14]. The risk stratification for AML patients was mainly based on the European LeukaemiaNet (ELN) classification [14–16]. The study was conducted in compliance with the provisions of the Declaration of Helsinki and approved by the Research Ethics Committee of Zhongnan Hospital at Wuhan University (Wuhan, China). Written informed consent was received from all patients and normal subjects before inclusion in the study and information was collected from the electronic patient record.

The haematopoietic stem cells (HSCs) and hyperleukocytic acute myeloid leukaemia cells were collected using a Fresenius COM.TEC machine from Zhongnan Hospital; the procedures were described in our previous reports [17]. The peripheral blood mononuclear cells (PBMCs) were separated with a Ficoll kit according to the manufacturer’s instructions (TBD, lot: LDS1075, China). Bone marrow mononuclear cells (MNCs) were collected from AML patients during bone marrow biopsy and separated according to the manufacturer’s instructions (TBD, lot: TBD2013CHU, China).

### Detection of oxidative stress levels

After AML cells were treated with CKI for 24 h, the collected AML cells were incubated with dichlorofluorescein diacetate (DCFH-DA) dye (Beyotime, No. S0033, China) for 25 min at 37 °C and then intracellular ROS levels were detected by flow cytometry (Cytomics FC 500, Beckman Coulter, USA). Cultured AML cells were collected to test T-AOC (No. A105), GSH (No. A006–1) and XOD (No. A002) with kits from the Nanjing Jiancheng Bioengineering Institute (Nanjing, China).

The T-AOC vitality, GSH content, H_2_O_2_ concentration (Beyotime, No. S0038, China) and CAT activity (Nanjing Jiancheng Bioengineering Institute, No. A007–1) in mice blood were tested according to the manufacturer’s instructions.

### Cell proliferation and cell apoptosis

Cell Counting Kit-8 (CCK8) assays were used to determine cell proliferation according to the manufacturer’s instructions (Dojindo, Lot.JE603). Briefly, 1 × 10^4^ AML cells per well were seeded into a 96-well plate and subsequently cultured with a different dose of CKI for 48 h. Then, 10 μl CCK-8 reagent was added to each well and the plate was incubated for the appropriate amount of time. Then, the absorbance was measured at 450 nm using a microplate reader (SpectraMax M2, Molecular Devices, China).

Cell apoptosis was measured using an Annexin V-FITC/PI apoptosis kit (MultiSciences (Lianke) Biotech Co., Ltd.) according to the manufacturer’s instructions. The detailed methods for the apoptosis, JC-1 and cell cycle assays are shown in the Additional file 3: Supplemental methods.

Apoptotic cell morphology was detected by transmission electron microscopy (TEM). U937 cells were prepared as described previously [18]. Briefly, the cells were fixed with 2.5% cold glutaraldehyde overnight at 4 °C and post-fixed in 1% osmium tetroxide for 2 h. The cells were then treated with different concentrations of acetone for dehydration and embedded in epoxy resin. Next, the samples were sectioned with an ultramicrotome (Leica, EM UC7, Germany), stained with uranium and lead, and observed using a transmission electron microscope (HT7700, Hitachi) at the Research Center of Medicine and Structural Biology at Wuhan University.

### Cytotoxicity assays

For drug toxic assays, the U937 cells were treated with eight different CKI dosages (14, 28, 42, 56, 70, 84, 98, and 112 μg/ml) for 48 h, and the inhibition rates were detected by MTT assay. The IC50 was calculated from a plotted graph with X representing the dosage and Y representing the inhibition rate in Excel. The cytotoxic effects on HSCs and T cells were examined by MTT assays to determine the cell death rate after CKI or Ara-c treatment for 48 h, respectively.

The cytotoxicity and cell pore-forming activities [19] were measured using a lactate dehydrogenase (LDH) release assay kit (Beyotime, NO. C0017) to examine the cell viability and cell lysis.

### Detecting the cytotoxicity at the single-cell microfluidics level

To detect the cytotoxicity of CKI at the single-cell level, microfluidics chips were designed to perform the injection and capture of single cells with optical technology [20–22]. Briefly, the clip size on an optofluidics chip was designed according to the cell size. AML cells (THP-1 cells, U937 cells, HL60 cells and human AML cells) were injected into the chip and fixed. CKI was injected from another inlet to treat the AML cells. The dynamic changes in the cell morphology after CKI treatment at different time points were observed by microscopy (NIKON, ECLIPSE TiU). The images were acquired with a sCMOS camera (Hamamatsu, ORCA-Flash 4.0 v2).

Molm-13 GFP+ cells were treated with 110 μl/ml CKI on optofluidics chips and the single-cell morphology were observed under a laser scanning confocal microscope (Nikon, A1R). The images were captured using NIS-Elements (AR 4.50.00). In a 6-well plate, 5 × 10^5^ Molm-13 GFP^+^ cells per well were treated with CKI for 48 h at 37 °C. Then, the fluorescence intensity changes of the cells were observed under a fluorescence microscope (OLYMPUS IX73) with intensity of 50 and a 121 ms exposure time.

### Dimethylation labelling reaction and hybrid quadrupole-TOF LC-MS/MS detection

The proteins were degraded to peptides by tryptic digestion as previously described. [23] Then, the peptides from two different samples were labelled with isotopomeric dimethyl labels as described previously [24, 25]. The dimethyl-labelled samples were analysed using a hybrid Quadrupole-TOF LC-MS/MS Mass Spectrometer (TripleTOF 5600, AB Sciex Instruments). The parameters were the same as those described previously [23]. The ProteinPilot™ 4.5 software (AB Sciex, Software revision number: 1656) was used to analyse the label-based quantitation of the peptides and protein identification was performed with the integrated Paragon™ search algorithm (revision number: 4.5.0.0, 1654) as described previously [26]. Query searches were conducted against a Uniprot human reference database (version: 201801.fasta). The detailed information is described in the Additional file 3: Supplemental methods.

### Western blot

For Western blot, the AML cells were lysed with RIPA containing PMSF to make cell lysates. In total, 30 μg of proteins measured using a BCA protein assay kit (Thermos, Waltham, MA, USA) were subjected to 15% SDS-PAGE for Western blot. After electrophoresis, the gels were transferred onto 0.22-μm PVDF membranes (Millipore Corp.) and the membranes were blocked with 5% BSA in 0.1% TBST (0.1% Tween-20 in 1 × TBS). The blot was probed with rabbit polyclonal antibodies against Prdx3 (1:500, USCNK) and Trx1 (1 μg/ml, Abcam), and mouse monoclonal antibodies against Prdx2 (1:2000, Protein-tech), as primary antibodies, overnight at 4 °C. The membranes were incubated with HRP-conjugated goat anti-rabbit IgG (diluted 1:5000, Protein-tech) and goat anti-mouse IgG (diluted 1:5000, Protein-tech) as secondary antibodies for 1 h at RT and detected with the Immobilon™ Western Chemiluminescent HRP Substrate (Millipore, Billerica, USA) using a Western Chemiluminescent Imaging System (Tanon 5200).

### Laser confocal microscopy

Molm-13 and U937 cells were separately seeded into 6-well culture plates. After CKI treatment for 48 h, the cells were fixed in 4% paraformaldehyde (PFA) in 1 × PBS for 15 min at room temperature. The cells were first pre-treated and then blocked in 1 × PBS with 0.5% Triton X-100 and 5% BSA for 1 h. Next, the samples were incubated with anti-Prdx3 (1:100), Prdx2 (1:100), or Trx1 (1:100) antibodies overnight at 4 °C, followed by incubation with secondary antibodies conjugated to AF647 or CF568 (1:400 dilution). The samples were then analysed with a laser confocal microscope (OLYMPUS IX83, UltraVIEW VoX) with a 640 nm or 560 nm pulse. The images were collected with the Volivity software containing Acquisition, Quantitation, and Visualization modules.

### Animal experiments

Five-week-old female B-NSG mice were obtained from Beijing Biocytogen Co., Ltd. (permission number: SCXK 2016–0004) and maintained in a pathogen-free animal facility in laminar airflow cabinets with a 12-h light/12-h dark schedule. The animals were fed an autoclaved rodent diet ad libitum. All procedures followed the institutional and national guidelines for the care and use of laboratory animals.

The AML animal model [27, 28] and patient-derived xenografts (PDX) [29, 30] were constructed as follows. First, Molm-13 GFP^+^ cells were cultured in RPMI1640 medium (GIBCO) supplemented with 10% foetal bovine serum (FBS, GIBCO). At day 0, B-NSG female mice (five-week old) were given a 2.5 Gy χ-ray dose for total body irradiation, followed by an intravenous injection of harvested Molm-13 GFP^+^ cells (3.6 × 10^5^ per mouse) or human AML patient cells (1.7 × 10^7^ per mouse) separated from a hyperleukocytic AML patient via tail vein within 24 h of irradiation.

The leukaemia animal model was successfully established after injection of the cells via routine blood tests and peripheral blood cell smear tests. The mice were randomly distributed into two groups. The CKI group was intraperitoneally injected (i.p.) with CKI (courtesy of the Shanxi Zhengdong Pharmaceutical Co. LTD., Z14021231, China) at a dose of 50 mg/kg/mouse (diluted with saline in a final volume of 200 μl) once a day for 10 days, while the control groups received saline with the same volume of 200 μl i.p. once a day. All mice were sacrificed and the harvested tissue samples were used for further analysis.

### Routine blood examination, smear analysis, and bone marrow biopsy

A routine blood examination was performed by a Coulter STKS automated blood cell analyser (Beckman Coulter, USA). The fluorescence intensity of the blood smear and bone marrow smear on slides was observed under a fluorescence microscope (OLYMPUS IX73). The blood smear and bone marrow smear were also stained via Wright’s staining and observed with a microscope under an oil immersion lens (OLYMPUS BX41) to measure the ratio of leukaemia cells and determine whether the AML models were successfully constructed. The immunophenotypes of CD117, CD45, CD33 and CD34 in mouse peripheral blood were also measured by flow cytometry.

For the bone marrow biopsy, bone marrow collected from mouse thighs was fixed in formalin and embedded in paraffin for bone marrow examination. The sections were sliced using a slicing machine (Leica RM2235). Immunohistochemistry was performed per our previously described method [23]. The sections were incubated with anti-Prdx3 (1:100, USCNK), anti-Prdx2 (1:100, Protein-tech), and anti-Trx1 (1:100, Abcam) antibodies in this study.

The images were obtained with the Aperio versa 10.3.37 software using an ultra-high-resolution microscopic imaging system (Leica microsystems, Germany) at the Medical Research Institute at Wuhan University.

### Bioinformatics and statistical analysis

The heat map was analysed with R code (V i386 3.0.1). The identified proteins showing changes were annotated by GO terms using BGI WEGO (http://biodb.swu.edu.cn/cgi-bin/wego/index.pl). The protein-protein interaction networks were constructed using STRING v9.1 (http://string-db.org/cgi/input.pl). For all statistical comparisons, the data were analysed using the parametric unpaired *t* test in GraphPad Prism (version 6.0). The survival curve was also constructed by SPSS 24.0. *p* < 0.05 was considered statistically significant. All results are presented as the mean ± standard error of the mean.

## Results

### Antioxidant CKI decreased intracellular ROS levels in AML cells

After treatment with CKI, the intracellular ROS levels in THP-1, Molm-13, HL60, U937 and human AML cells were significantly reduced compared with controls (Fig. 1a). The total antioxidant capacity (T-AOC) and glutathione (GSH) contents increased in the supernatant of all five AML cells when treated by CKI (Fig. 1b, c). In homogenate, the T-AOC activity and GSH contents significantly increased in HL60 cells, Molm-13 cells and human AML cells, but there was no changed in THP-1 cells and had a slightly increase trendency in U937 cells. The xanthine oxidase (XOD) activity decreased in U937 cells and HL60 cells (Fig. 1d). Additionally, the superoxide dismutase (SOD) vitality was enhanced in the cultured U937 cells and HL60 cells (Additional file 4: Figure S2).

### CKI cytotoxicity on AML cells

The antioxidant CKI could markedly inhibit THP-1, U937, HL60 and Molm-13 cell proliferation (Fig. 2a). Additionally, the cell proliferation of human AML cells, which were separated from AML patients, was significantly inhibited after CKI treatment, including hyperleukocytic leukaemia (HLL) in AML (Fig. 2b). The cytotoxicity and cell pore-forming activity of U937, HL60, Molm-13 and human AML cells increased when treated with different concentrations of CKI (Fig. 2c, d). We tested the half maximal inhibitory concentration (IC50) of CKI on U937 cells; the IC50 value was 0.69 mg/ml (Fig. 2e). We then evaluated the cytotoxicity on normal cells including HSCs and T cells. We found that cytosine arabinoside (Ara-c) significantly promoted cell death with an IC50 value of 2.5 μg/ml, but it was low-toxic to normal cells for CKI (Fig. 2f). Cell count assays indicated that THP-1, U937, HL60 and HLL cell growth was significantly slowed by CKI treatment, while the healthy CD34+ and HSC CD34+ cells were not influenced by CKI (Additional file 5: Figure S3).

### Validation of the cytotoxicity of CKI on the single-cell level

**Fig. 3:**
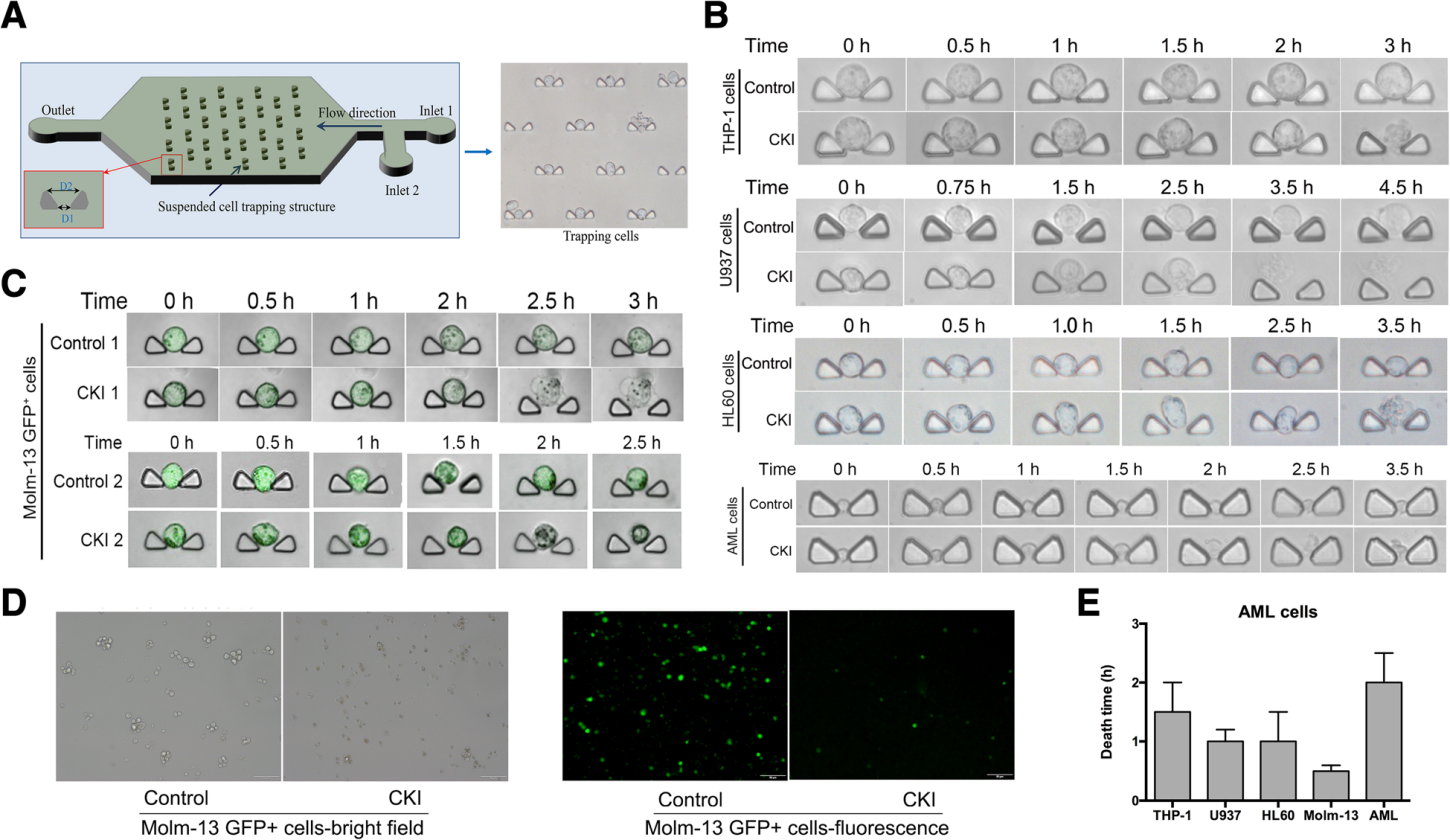
The variation in cell morphology observed with the optofluidics chips at the single-cell microfluidics level. **a** Optofluidics chip used for cell cytotoxic analysis. For AML cell lines, D1 = 7 μm and D2 = 35 μm. For AML patient cells, D1 = 5 μm and D2 = 30 μm. **b** The cell motion morphology of AML cells was observed after 110 μl/ml CKI treatment at the single-cell level on the microfluidics chip. AML: AML patient cells (PC) 2#. **c** Molm-13 GFP+ cells were treated with 110 μl/ml CKI on optofluidics chips and observed under a laser scanning confocal microscope. Upper line: pyroptosis. Bottom line: apoptosis. **d** Molm-13 GFP+ cells were treated with 110 μl/ml CKI for 48 h and observed under an OLYMPUS IX73 fluorescence microscope. Magnification of the objective lens: × 20. **e** The response time for AML cell death

We designed different optofluidics chips containing different sized clips according to cell size. A schematic diagram shows the AML cells injected into the chip and trapped on the clips (Fig. 3a). Compared to the control, THP-1 cells gradually shrunk, while the U937, HL60 and human AML cells became swollen and their membranes ruptured after CKI injection into another inlet on the optofluidics chips (Fig. 3b). The Molm-13 GFP^+^ cells displayed pore-forming or apoptotic morphologies and a weak fluorescence intensity, which were observed by confocal microscopy at the single-cell level (Fig. 3c). The cell morphology of the Molm-13 GFP+ cells was perforated and showed cell swelling and lysis. It is possible that pyroptosis occurred [19, 31] after CKI treatment, which is another type of programmed cell death that differs from apoptosis. At the group level, the Molm-13 GFP^+^ cells died in the bright field, and the fluorescence intensity was reduced by fluorescence microscopy after for 48 h of treatment with CKI (Fig. 3d). The death time for a single cell from four AML cell lines and one AML patient cell type was analysed in Fig. 3e. Molm-13 GFP^+^ cells died less than 1 h after CKI treatment, which was the fastest response time and even more sensitive to CKI stimulation than other cells.

### Antioxidant CKI promoted AML cell apoptosis

**Fig. 4:**
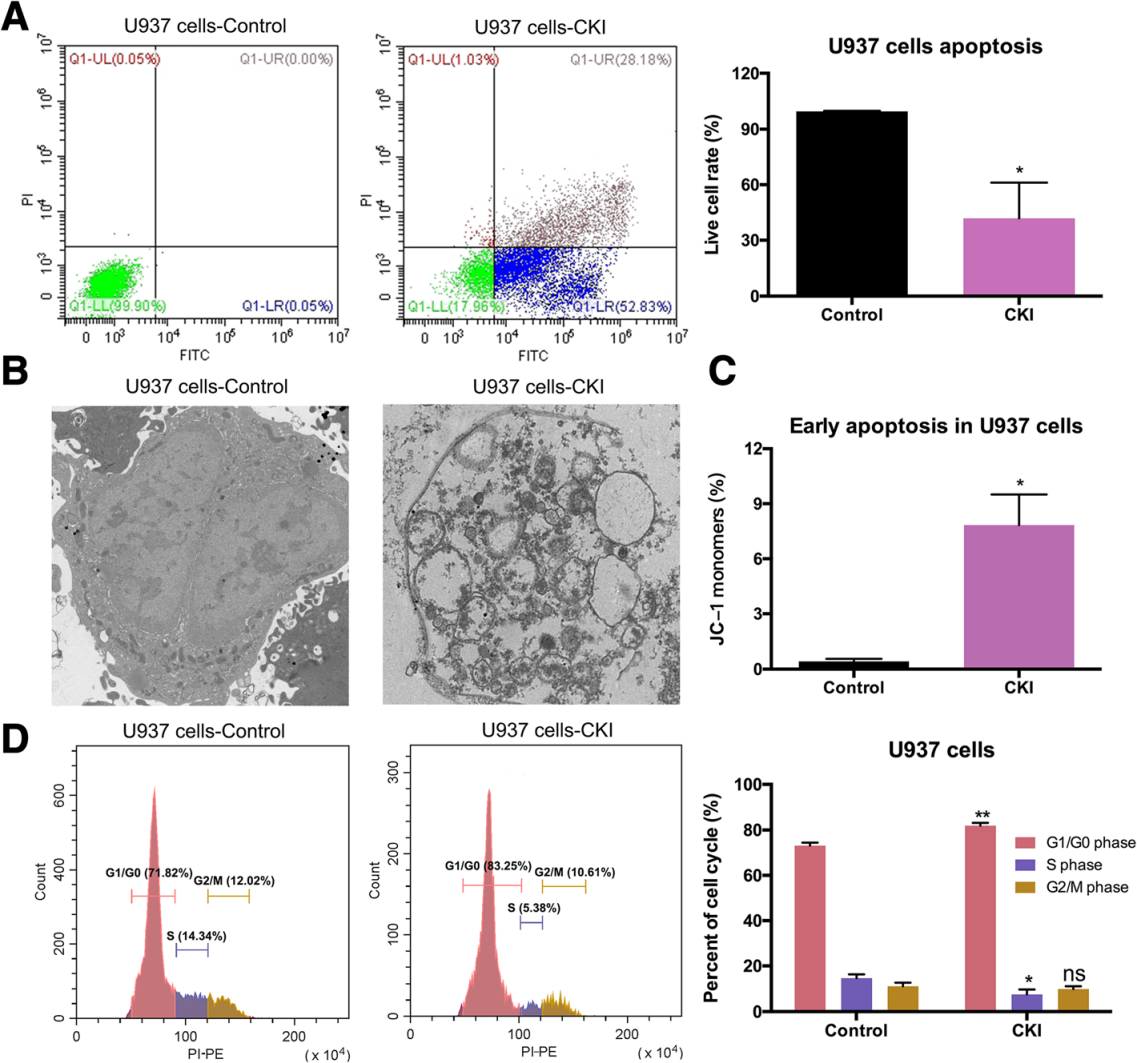
Cell apoptosis and cell cycle analysis in U937 cells. **a** Apoptosis was tested by flow cytometry after U937 cells were treated with 110 μl/ml CKI for 48 h. **b** The morphological apoptotic characteristics were observed by TEM after U937 cells were treated with 110 μl/ml CKI for 48 h. Magnification: × 2000. **c** Early apoptosis was determined by JC-1 assay after U937 cells were treated with 110 μl/ml CKI for 24 h. **d** Cell cycle was analysed by flow cytometry after U937 cells were treated with CKI for 48 h

Flow cytometry showed that CKI induced apoptosis in U937 cells (Fig. 4a). Apoptotic vacuolization was clearly visible in U937 cells. The apoptosis process was accelerated after CKI treatment and observed by TEM, while the cells in the control group continued dividing (Fig. 4b). JC-1 monomers accumulated, indicating decreased mitochondrial membrane potential, and early apoptosis occurred in U937 cells when treated with CKI (Fig. 4c). CKI also promoted the apoptosis of HL60 and THP-1 cells as shown in Additional file 6: Figure S4. Additionally, U937 cells were arrested in G1/G0 phase after CKI treatment (Fig. 4d).

### Prdx3 was identified by LC-MS/MS after CKI against AML

**Fig. 5:**
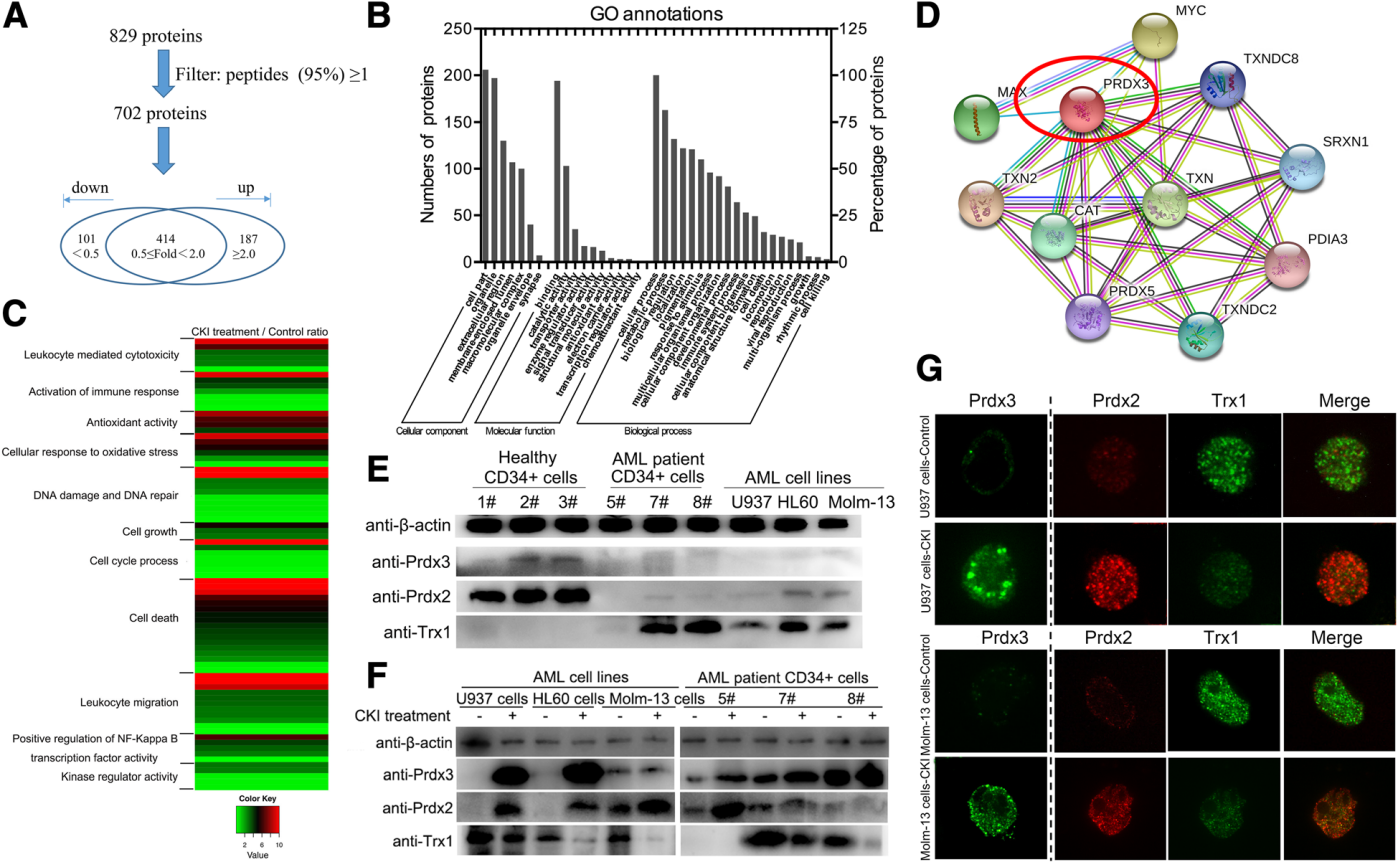
Prdx3 was identified by LC-MS/MS for CKI against AML, and CKI regulated Prdxs/Trx1 in AML. **a** Data analysis. **b** GO annotation with 288 differential expressed proteins. **c** Heat map showed the changes in 11 functions in 54 differential expressed proteins after CKI treatment. **d** The interaction between PRDX3 and other selected proteins by STRING. **e** Validation of Prdx3, Prdx2, and Trx1 protein expression in healthy and AML cells by Western blot. **f** The protein expression changes to Prdx3, Prdx2, and Trx1 after treatment of AML cells with CKI and their detection by Western blot. **g** The expression and interaction of Prdx3, Prdx2, and Trx1 after CKI treatment in AML cells and their observation using laser confocal microscopy

To understand the mechanism for the anti-leukaemia effects of CKI, we analysed the differentially expressed proteins (DEPs) through dimethyl labelling quantitative proteomics in U937 cells. The proteins from two groups were digested and labelled with two different dimethyl labelling reagents to allow for quantitation and identification by LC-MS/MS. The workflow is shown in Additional file 7: Figure S5. A total of 829 proteins were identified, and the protein list was filtered based on at least one peptide with 95% confidence. After filtering, a total of 288 proteins were differentially expressed, of which 101 proteins were down-regulated with fold change < 0.5 and 187 proteins were up-regulated with fold change ≥2.0 from our MS detection results (Fig. 5a). Detailed information on the proteins identified by MS is listed in Additional file 8: Table S2.

The 288 DEPs were further annotated by Gene Ontology (GO) analysis. All DEPs were classified according to their biological process, cellular component and molecular function (Fig. 5b), and most of these proteins were found to be involved in binding, catalytic activity, metabolism, biologic regulation, stimuli response, and immunity. Fifty-four proteins involved in eleven related functions, including leukocyte mediated cytotoxicity, activation of immune response, antioxidant activity, cellular response to oxidative stress, DNA damage and DNA repair, cell growth, cell cycle process, cell death, leukocyte migration, positive regulative of NF-Kappa B, transcription factor activity, and kinase regular activity, were analysed by heat map (Fig. 5c). The 54 proteins are listed in Additional file 9: Table S3. Peroxiredoxins (Prdxs) are a family of proteins that function as antioxidants in cells [32]. It has been reported that Prdx3 is a mitochondrial protein that decreases mtROS levels [33, 34]. In our MS detection data, the expression of Prdx3 was 6.5-fold greater in U937 cells than in the control after CKI treatment and was selected for further analysis due to its antioxidant activity. The proteins that interacted with Prdx3 were analysed by STRING v9.1 and are shown in Fig. 5d.

### Antioxidant CKI regulated the Prdxs/Trx1 in AML

Peroxiredoxin-2 (Prdx2) is another Prdxs family member located in cytoplasm that reduces intracellular ROS level. It has been reported that the tumour suppressor gene *Prdx2* is epigenetically silenced in AML and causes the generation and accumulation of ROS, which promotes AML cell proliferation [1]. Moreover, the Prdx2 could catalyse Trx1 [35], and Prdx2 was interacted with Trx1, which was shown by STRING software in Additional file 10: Figure S6.

In this study, we detected the Prdx3 and Prdx2 proteins that resisted ROS levels. QPCR results showed that their mRNA levels were significantly increased in CKI-treated AML cell lines and AML patient cells (Additional file 11: Figure S7). The Prdx3, Prdx2 and Trx1 expression levels were confirmed by immunoblotting. In AML cell lines or patient cells, Prdx2 and Prdx3 protein expression was lower than in healthy controls, while Trx1 levels were higher than in healthy controls (Fig. 5e). After CKI treatment, Prdx3 and Prdx2 protein expression levels increased while Trx1 levels decreased (Fig. 5f). The expression of the three proteins were demonstrated by laser confocal microscopy. As shown in Fig. 5g, Prdx3 and Prdx2 were expressed higher and Trx1 was reduced in Molm-13 and U937 cells. The up-regulation in Prdx3 expression is in agreement with our mass spectrometry and Western blot findings. Additionally, Prdx2 was interacted with Trx1 after CKI treatment (Fig. 5g).

### Anti-leukaemia effects of CKI in vivo in B-NSG mice

The anti-leukaemic effects of CKI were verified by construction of an AML animal model via a Molm-13 GFP+ cells injection. The construction of the AML model and administration of CKI to irradiated B-NSG mice (*n* = 10, 5 mice per group) are shown in Additional file 12: Figure S8a. The AML animal model was successfully constructed and was detected by blood smear and bone marrow smear (Additional file 12: Figure S8b). The fluorescence was observed in the blood smear at day 8. At day 10, we collected bone marrow from a dead mouse and bone marrow smear analysis was performed; we found that the Molm-13 GFP+ cells were targeting the bone marrow. When all the mice were euthanized on the 29th day, the bone marrow smears were stained with Wright’s staining and the acute myeloid leukaemia cells were observed by microscopy under an oil immersion lens. The body weights of mice in the CKI group were quickly restored compared to the saline group two weeks after the construction of the AML model (Additional file 12: Figure S8c). Twenty-nine days after AML cell inoculation, the survival rate was better than the saline group (Additional file 12: Figure S8d). Heart, spleen, lung and kidney tissues in the saline group were swollen compared with the CKI group (Additional file 12: Figure S8e), though the tissues’ weight indexes showed no significant differences.

**Fig. 6:**
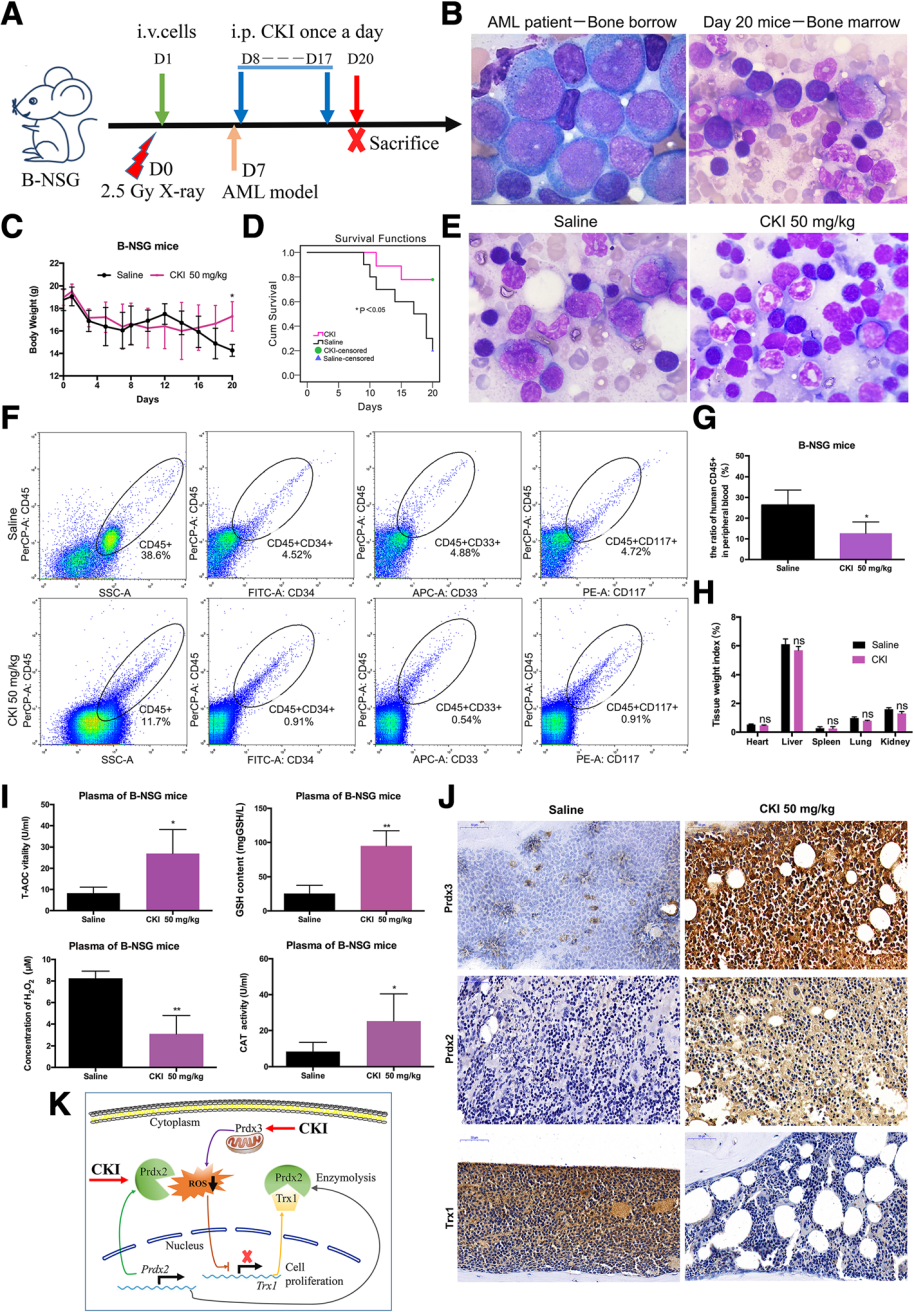
Therapeutic efficacy of CKI on the patient-derived xenograft model of acute myeloid leukaemia. **a** The schematic diagram for the AML PDX model. **b** Analysis of bone marrow smear. At day 20, the bone marrow smear was stained and leukaemia cells were observed. Magnification fold: × 100 under an oil immersion lens. **c** The changes in body weight. **d** The survival analysis. **e** The remission state of bone marrow in the CKI group. **f** The immunophenotype of leukaemia cells in peripheral blood by flow cytometry analysis. **g** Statistics for the ratio of leukaemia cells. **h** The tissue weight index. **i** Testing the T-AOC vitality, GSH content, H_2_O_2_ concentration and CAT activity in plasma from B-NSG mice. **j** Detection of Prdx3, Prdx2, and Trx1 expression by IHC in the PDX model. **k** The model for the anti-leukaemic effects of CKI

We also constructed an AML patient-derived xenograft (PDX) model of acute myeloid leukaemia to investigate the anti-leukaemic effects of CKI. The B-NSG mice (*n* = 19) were injected with human hyperleukocytic AML patient cells. The construction of the PDX model and CKI injection into B-NSG mice are shown in Fig. 6a (Saline group: n = 10; CKI group: *n* = 9). Among the two AML animal models, the PDX AML animal model mice died faster, so they could not be observed for 29 days. At the 20th day, all mice were euthanized and acute myeloid leukaemia cells in the bone marrow smear were observed, which indicated that the PDX model was successfully constructed (Fig. 6b). After CKI injection, the body weights of the mice in the CKI group were gradually restored, while the body weights of the mice in the saline group declined (Fig. 6c). The survival rate in the CKI group was better than in the saline group for the AML PDX model (Fig. 6d), and the mice in the CKI group were in a state of remission (Fig. 6e). The ratio of human leukaemia cells in peripheral blood decreased after CKI injection (Fig. 6f, g). Additionally, the heart, spleen, lung and kidney tissues in the saline group were swollen compared with the CKI group (Fig. 6h), though the tissues’ weight indexes showed no significantly differences.

Moreover, in blood plasma, the T-AOC vitality and GSH contents in the CKI group were markedly higher than in the saline group, and the CAT activity increased while the H_2_O_2_ concentration significantly reduced in the CKI group (Fig. 6i). The bone marrow biopsy results showed that the expression levels of Prdx3 and Prdx2 increased and Trx1 decreased, as detected by immunohistochemistry in the AML PDX model CKI group (Fig. 6j), which is consistent with the cell experiment results.

We provided a model for the anti-leukaemic effects of CKI (Fig. 6k). CKI decreased intracellular ROS levels by up-regulating the expression of Prdx2 in the cytoplasm and Prdx3 in the mitochondria and down-regulating Trx1 expression, which maintained the intracellular REDOX and further inhibited AML cell proliferation.

## Discussion

In this study, we reported that the compound kushen injection (CKI) is effective for treatment AML by regulating the ROS signalling pathway. CKI inhibited the proliferation of non-hyperleukocytic not only AML cells but also hyperleukocytic AML cells, even at the single-cell level, which indicated that CKI may be used for the treatment of hyperleukocytic AML patients after leukapheresis. Additionally, as we have already known, after chemotherapy, leukaemia stem cells (LSCs) and residual lesions are present. LSCs showed relatively low levels of ROS [36], but external environment such as air pollution, ray irradiation and chemical reagents increase ROS levels and lead to AML disease relapse [7, 37]; therefore, CKI may also be a good antioxidant and have the potential to control ROS levels to prevent AML relapse.

ROS production and the peroxidase defence system are regulated and balanced and closely related to apoptosis and necrosis. The peroxiredoxins (Prdxs) family resists ROS and scavenges free oxygen radicals in the body, protecting intracellular genomic DNA, lipids and proteins from oxidative damage by regulating intracellular ROS levels. In an excessive H_2_O_2_environment, Prdx is irreversibly transformed into a highly oxidized derivative form (Prdx-SO3), which cannot maintain homeostasis levels of intracellular oxidative stress [38].

Mitochondria are the main source of intracellular ROS production. In mitochondria, Prdx3 is mainly involved in H_2_O_2_ detoxification and is used to regulate ROS levels. In mammals, Prdx-3 only accounts for 1.6% of soluble mitochondrial protein, but it is responsible for the detoxification of 90% of H_2_O_2_ [39]. Under oxidative stress conditions, PRDX3 is recruited to UNG1 binding to mtDNA and combines with UNG1 through a disulphide bond, protecting UNG1 from degradation and preventing oxidative damage to mitochondrial DNA to enhance the cell resistance to oxidative stress [40]. Studies have reported that the deletion of PRDX3 resulted in increased H_2_O_2_ in the mitochondria [41], and the levels of mitochondrial ROS in THP-1 cells with the PRDX3 gene knocked out was significantly higher than in controls [33]. We have identified the increase in Prdx3 expression after CKI treatment by quantitative proteomics and have verified this increase in expression by qPCR, Western blot and immunohistochemistry.

Prdx2 is a Cys-dependent peroxidase that located in the cytoplasm. Agrawal-Singh et al. proposed that Prdx2 is a newly discovered tumour suppressor gene in AML. Histone H3 acetylation decreased and methylation increased on the Prdx2 promoter, leading to lower Prdx2 expression and the accumulation of ROS, which further promoted the malignant proliferation of AML cells and poor prognosis in AML patients. When Prdx2 expression was up-regulated, ROS levels decreased and AML cell growth was inhibited [1]. In our study, CKI could promote Prdx2 and Prdx3 expression to exert detoxification effects on H_2_O_2_ or free oxygen radicals and regulate the REDOX state to control AML cell growth. However, whether CKI can regulate Prdx2 methylation and acetylation levels and affect Prdx2 expression is worth further investigation.

Trx1 is a small molecular protein that plays important physiological functions, such as protein binding and transcriptional regulation [42, 43], and has an important role in maintaining the stable REDOX state. High-expression Trx1 promoted tumour cell growth by influencing the tumour microenvironment and regulating DNA synthesis and transcription factors [44]. The overexpression of the *Trx1* gene activated hypoxic induction factor 1 (HIF-1α) in cancer cells, increasing VEGF expression [45, 46] and promoting tumour angiogenesis, which leads to tumour invasion and metastasis [47]. Overexpressed Trx1 was involved in DNA damage by stabilizing p21 expression and being associated with haematological system diseases [48]. Following inhibition of Trx1 expression, leukaemia cell cycle was blocked [49]. Trx1 is located in cytoplasm, and the activation region for the REDOX centre at the C-terminal is Cys-Gly-Pro-Cys [50]. Trx1 is a Prdx2 substrate and involved in maintaining the cell REDOX steady state [35]. As mentioned above, we provided a potential molecular mechanism for CKI against AML. CKI decreased the levels of intracellular ROS by up-regulating Prdx2 expression in the cytoplasm and Prdx3 expression in the mitochondria and down-regulating Trx1 expression. Perhaps, higher Prdx2 expression catalyses Trx1 enzymolysis to maintain intracellular REDOX and further inhibit AML cell proliferation.

## Conclusion

CKI inhibited intracellular ROS levels in AML cells by increasing Prdx2 and Prdx3 expression and decreasing Trx1 expression. The antioxidant CKI inhibits cell proliferation by regulating the ROS signalling pathway in AML and is a promising drug for the treatment of AML in the clinic. We aimed to explore the therapeutic efficacy of low-toxic natural antioxidants against AML by regulating ROS pathways and providing new strategies to improve survival in AML patients.

## Additional files

Additional file 1: **Figure S1.** The component of CKI. (JPG 68 kb)

Additional file 2 **Table S1.** The information of new diagnosed AML patients. (XLSX 35 kb)

Additional file 3: Supplemental methods. (DOCX 23 kb)

Additional file 4: **Figure S2.** The SOD vitality after CKI treatment. (TIFF 518 kb)

Additional file 5: **Figure S3.** The cell growth. Cell growth was tested by trypan blue staining to analyse the cell count after 110 μl/ml CKI treatment for 48 h. (TIF 882 kb)

Additional file 6: **Figure S4.** Cell apoptosis and cell cycle for HL60 cells and THP-1 cells. (A) Cell apoptosis was tested by flow cytometry after HL60 cells were treated with 110 μl/ml CKI for 48 h. (B) Cell cycle was analysed by flow cytometry after HL60 cells were treated with CKI for 24 h. (C) Early apoptosis was determined by JC-1 assay after THP-1 cells were treated with 110 μl/ml CKI for 24 h. (TIF 2472 kb)

Additional file 7: **Figure S5.** Workflow for the quantitative proteomics with dimethylation labelling after U937 cells were treated with CKI. (JPG 198 kb)

Additional file 8: **Table S2.** The identified proteins by LC-MS/MS. (XLSX 74 kb)

Additional file 9: **Table S3.** The identified 54 differentially expressed proteins. (XLSX 28 kb)

Additional file 10: **Figure S6.** The proteins interacting with Prdx2 by STRING analysis. (TIF 1604 kb)

Additional file 11: **Figure S7.** The mRNA expression levels of Prdx2 and Prdx3 after CKI treatment. (TIFF 785 kb)

Additional file 12: **Figure S8.** The anti-leukaemic effects of CKI on B-NSG mice with Molm-13 GFP+ cell injections. (A) A schematic diagram of the AML animal model. (B) Analysis of the blood and bone marrow smears. At day 8, the blood was collected from the tail vein using a capillary tube at day 8 after injection of the Molm-13 GFP+ cells and blood smears were performed. At day 10, bone marrow smear analysis was performed to detect Molm-13 GFP+ cell targeting. The fluorescence intensity was observed by fluorescence microscopy. Magnification fold: ×20. At day 29, the bone marrow smears were stained and leukaemia cells were observed. Magnification fold: ×100 under an oil immersion lens. (C) The changes in body weight. (D) The survival analysis. (E) The tissue weight index. (TIF 4232 kb)

## Abbreviations

8-oH-dG: 8-hydroxydeoxyguanosine
AML: Acute myeloid leukemia
Ara-c: Cytosine arabinoside
CKI: Compound kushen injection
GSH: Glutathione
HLL: Hyperleukocytic leukaemia
HSCs: Haematopoietic stem cells
Jab1: c-Jun activation domain-binding protein l
LDH: Lactate dehydrogenase
NOX: NADPH oxidase
PBMCs: Peripheral blood mononuclear cells
PDX: Patient-derived xenograft
Prdx2: Peroxiredoxin-2
Prdx3: Peroxiredoxin-3
ROS: Reactive oxygen species
T-AOC: Total antioxidant capacity
TEM: Transmission electron microscopy
Trx1: Thioredoxin 1
XOD: Xanthine oxidase
