# Supplementary figures and images for: Compound kushen injection suppresses human acute myeloid leukaemia by regulating the Prdxs/ROS/Trx1 signalling pathway
Source: J Exp Clin Cancer Res. 2018 Nov 19;37:277. doi: 10.1186/s13046-018-0948-3 (PMC6245615; doi:10.1186/s13046-018-0948-3)

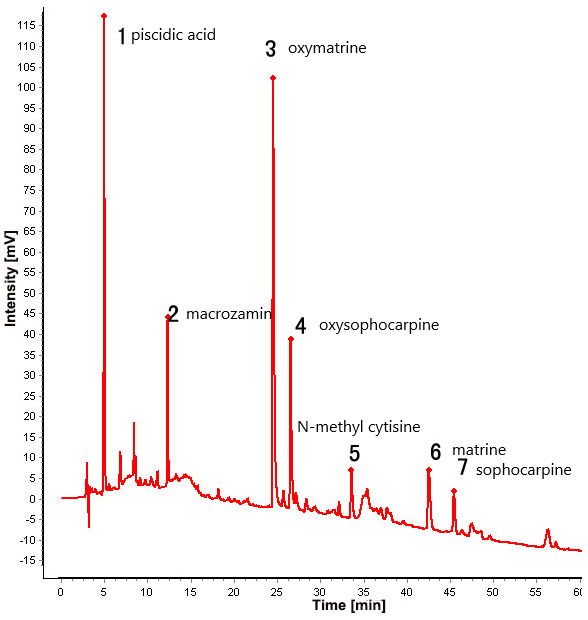

Supplement: Supplementary file 1 — Figure S1. The component of CKI. (JPG 68 kb) [file 13046_2018_948_MOESM1_ESM.jpg]

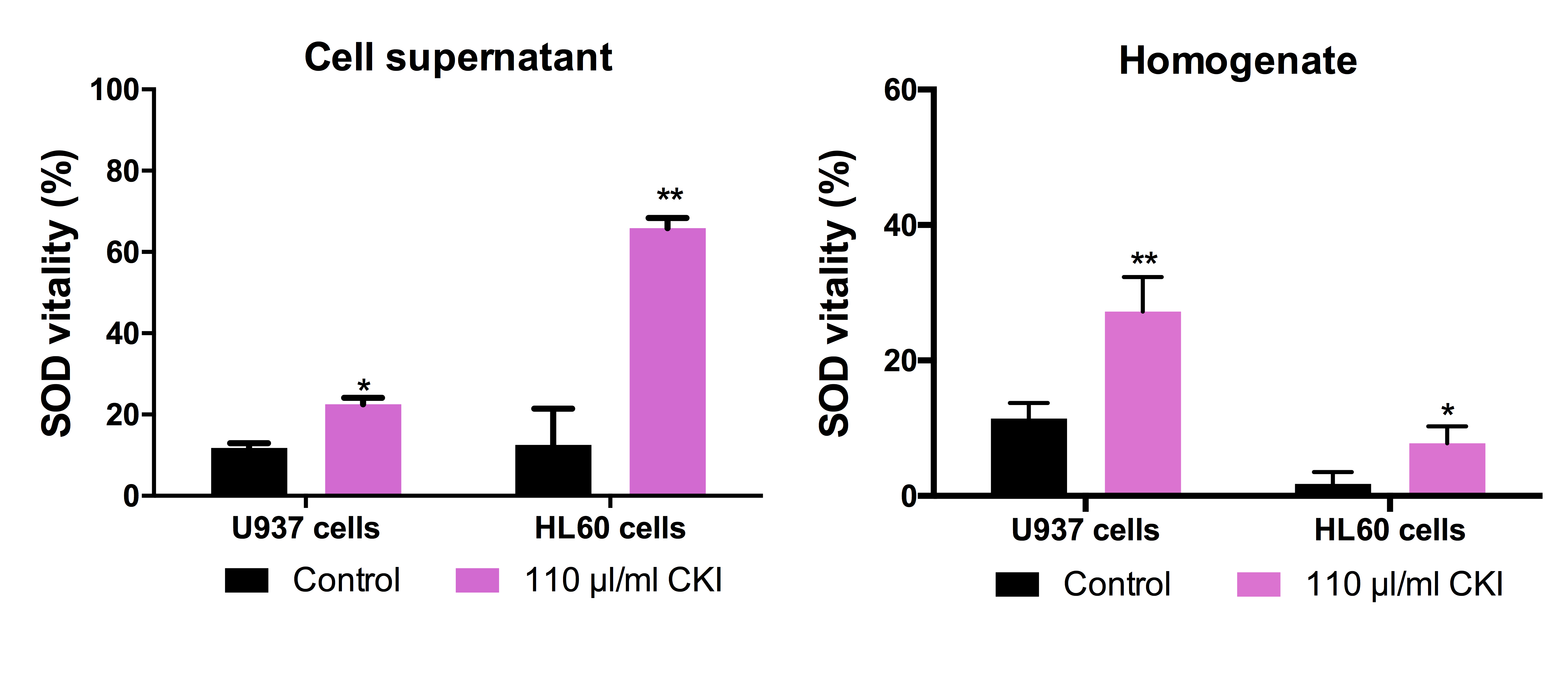

Supplement: Supplementary file 4 — Figure S2. The SOD vitality after CKI treatment. (TIFF 518 kb) [file 13046_2018_948_MOESM4_ESM.tiff]

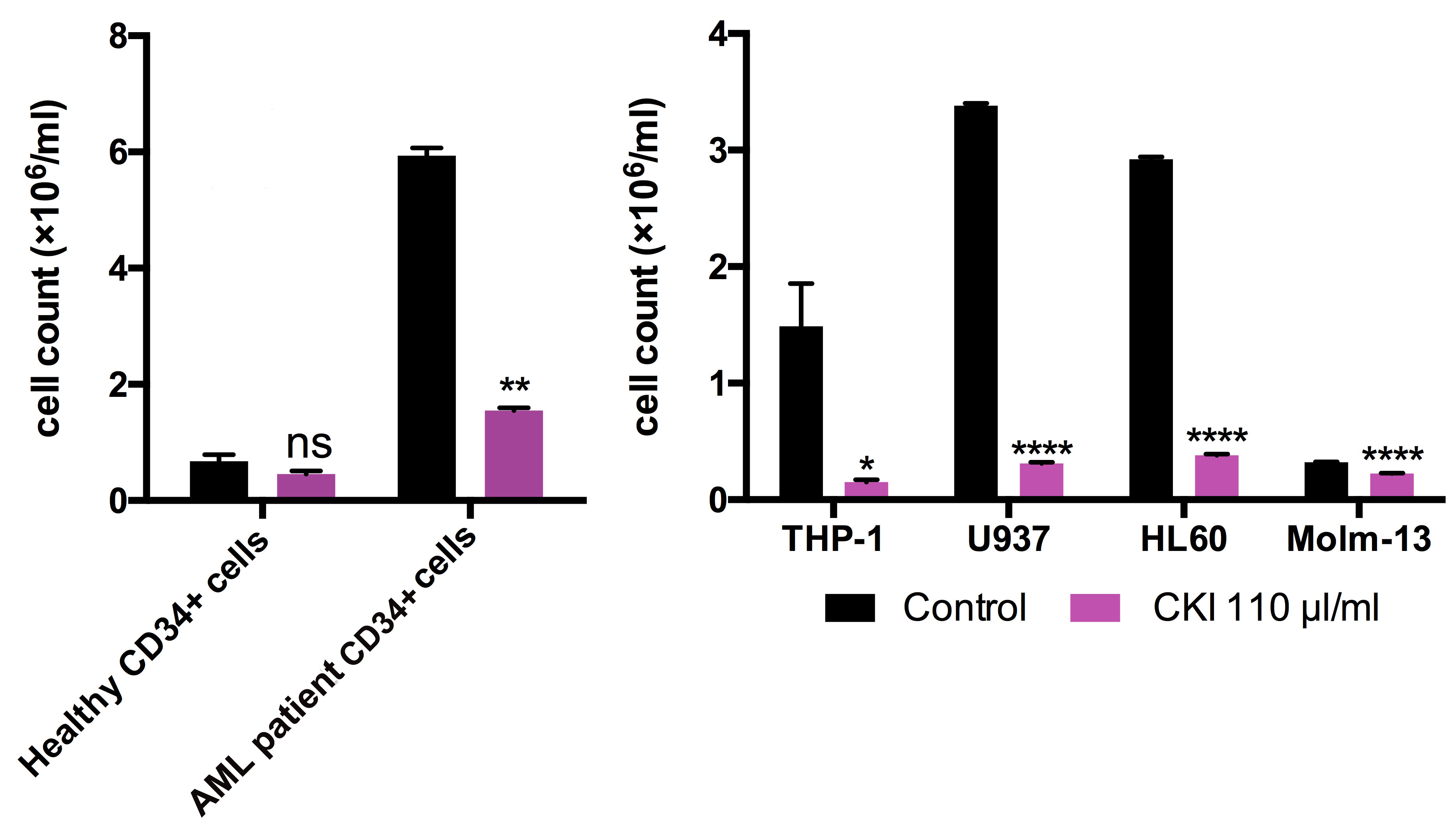

Supplement: Supplementary file 5 — Figure S3. The cell growth. Cell growth was tested by trypan blue staining to analyse the cell count after 110 μl/ml CKI treatment for 48 h. (TIF 882 kb) [file 13046_2018_948_MOESM5_ESM.tif]

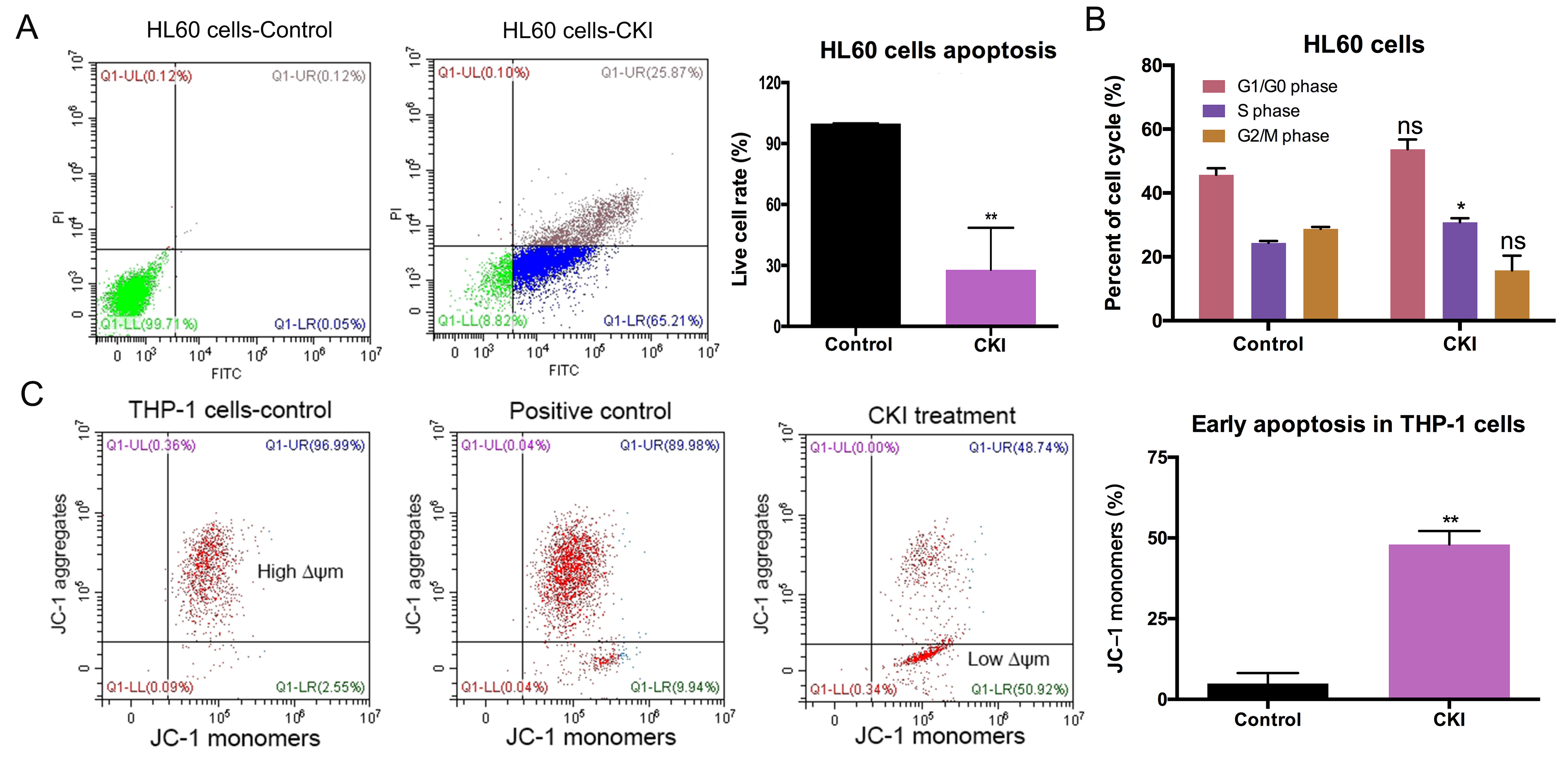

Supplement: Supplementary file 6 — Figure S4. Cell apoptosis and cell cycle for HL60 cells and THP-1 cells. (A) Cell apoptosis was tested by flow cytometry after HL60 cells were treated with 110 μl/ml CKI for 48 h. (B) Cell cycle was analysed by flow cytometry after HL60 cells were treated with CKI for 24 h. (C) Early apoptosis was determined by JC-1 assay after THP-1 cells were treated with 110 μl/ml CKI for 24 h. (TIF 2472 kb) [file 13046_2018_948_MOESM6_ESM.tif]

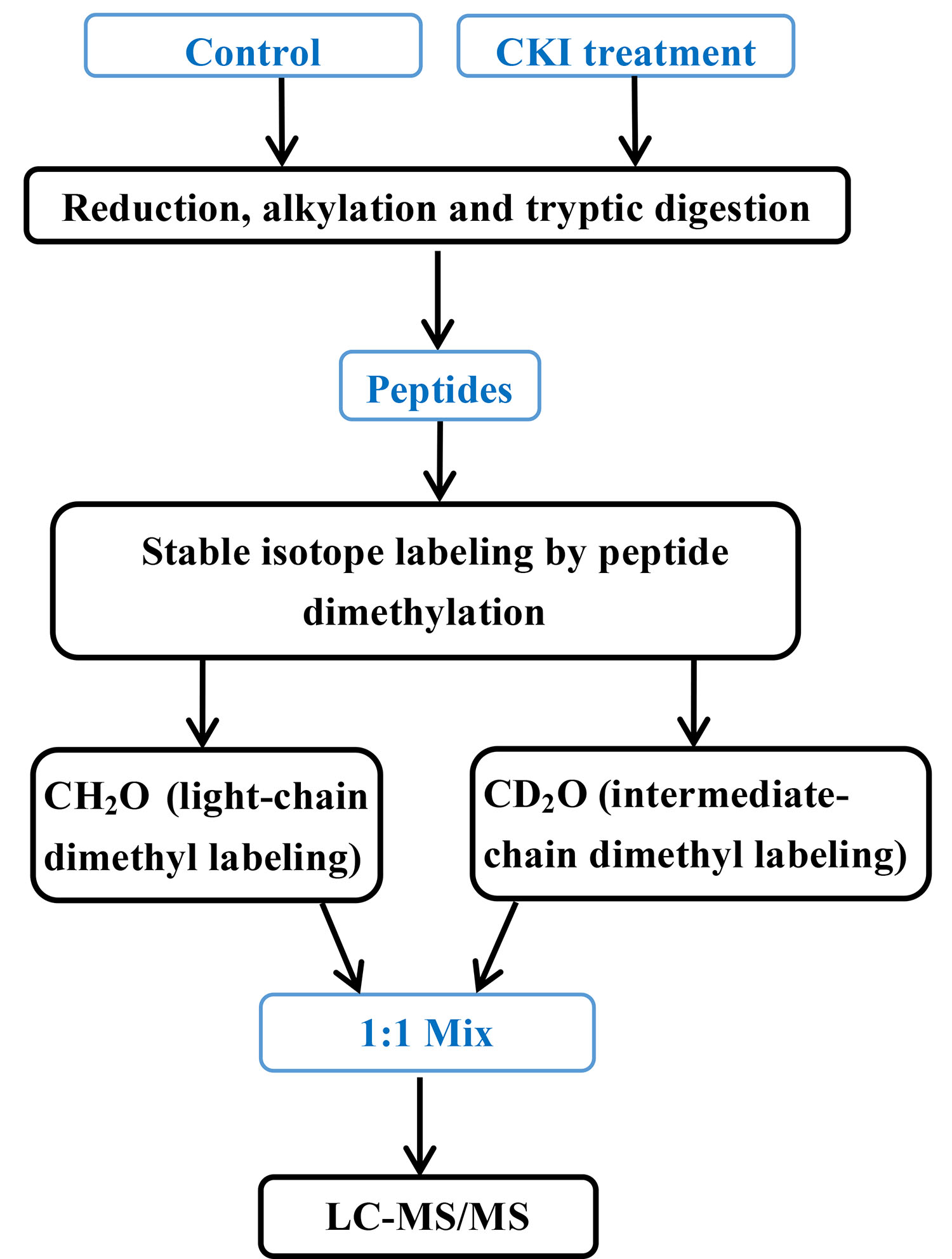

Supplement: Supplementary file 7 — Figure S5. Workflow for the quantitative proteomics with dimethylation labelling after U937 cells were treated with CKI. (JPG 198 kb) [file 13046_2018_948_MOESM7_ESM.jpg]

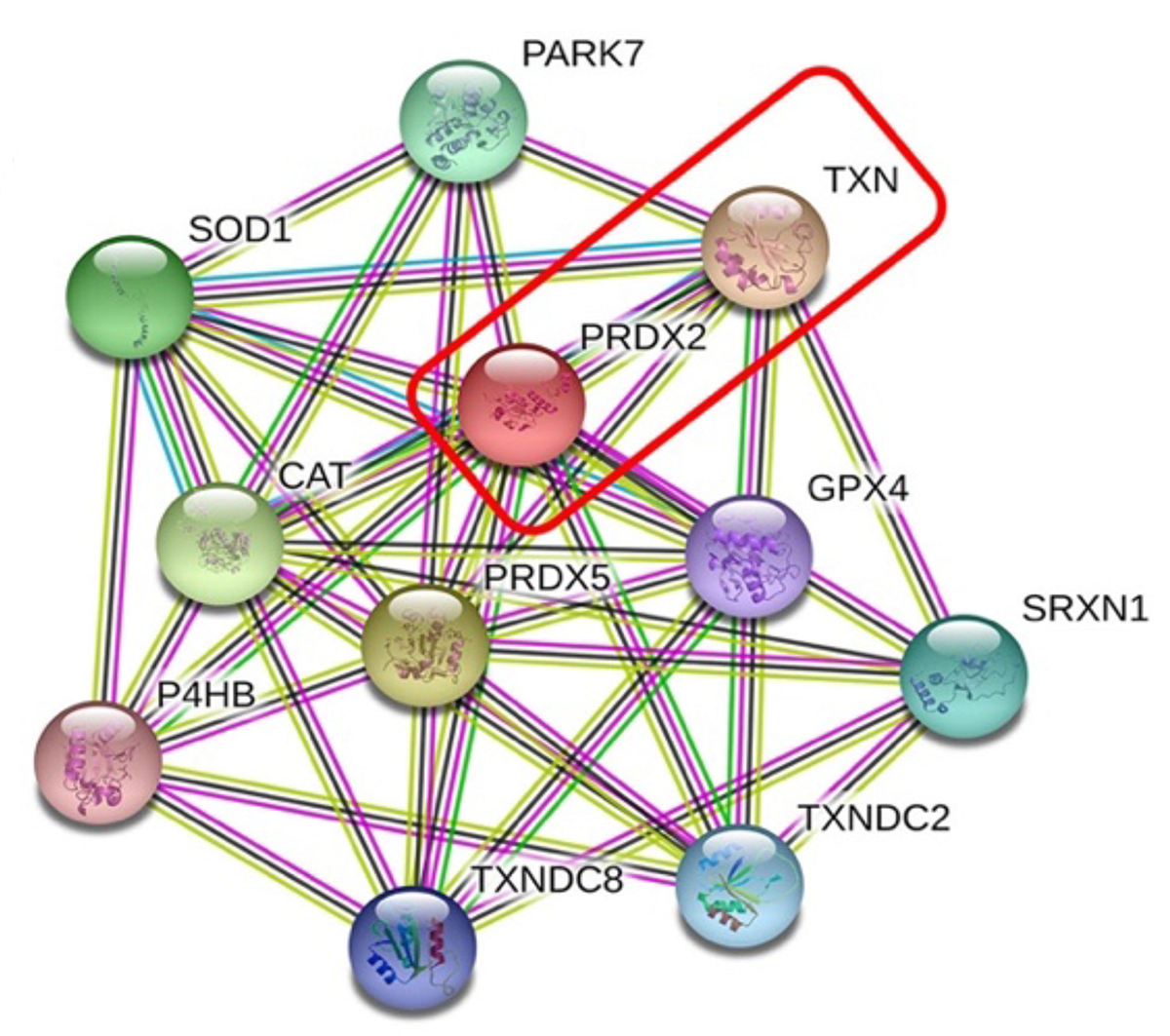

Supplement: Supplementary file 10 — Figure S6. The proteins interacting with Prdx2 by STRING analysis. (TIF 1604 kb) [file 13046_2018_948_MOESM10_ESM.tif]

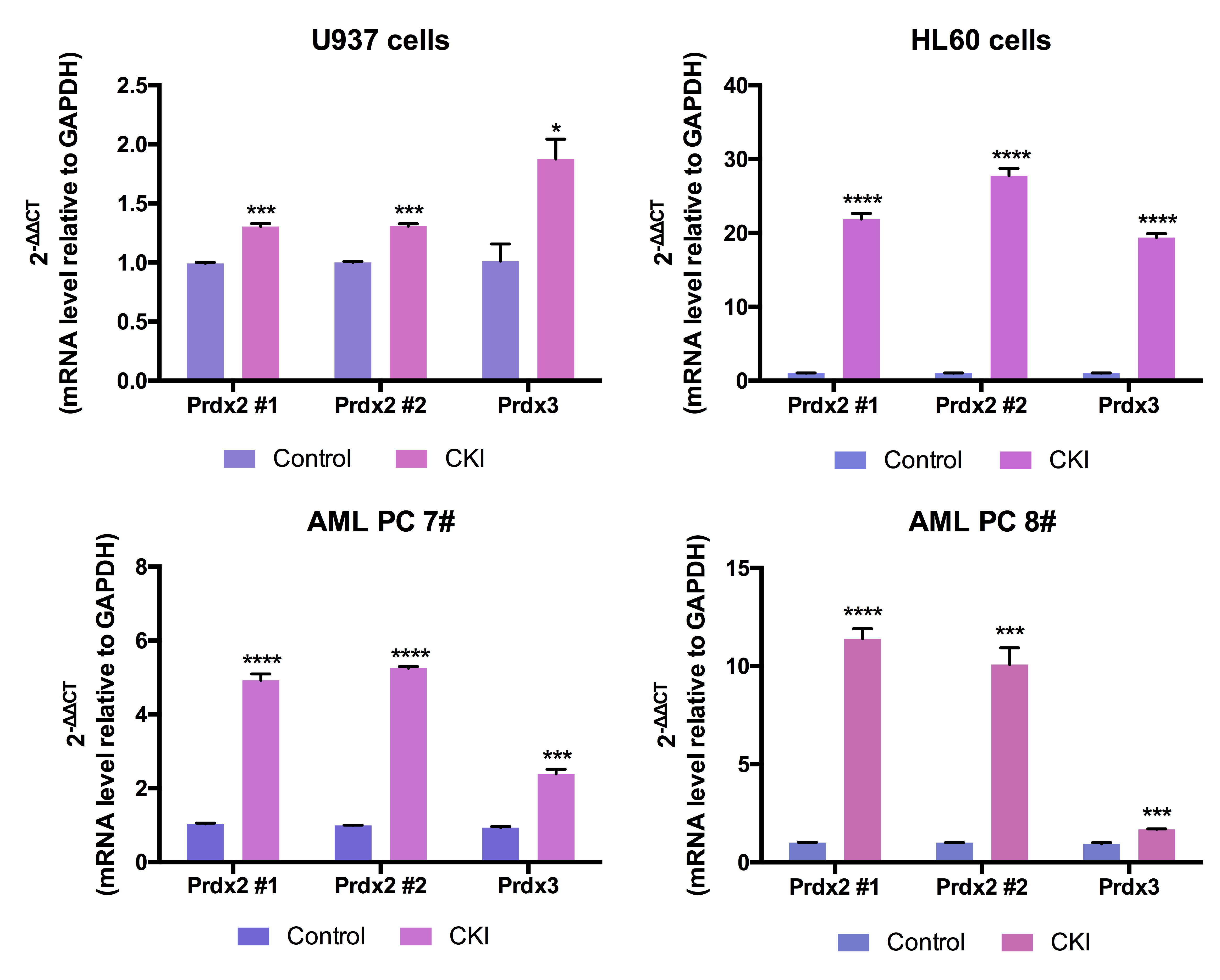

Supplement: Supplementary file 11 — Figure S7. The mRNA expression levels of Prdx2 and Prdx3 after CKI treatment. (TIFF 785 kb) [file 13046_2018_948_MOESM11_ESM.tiff]

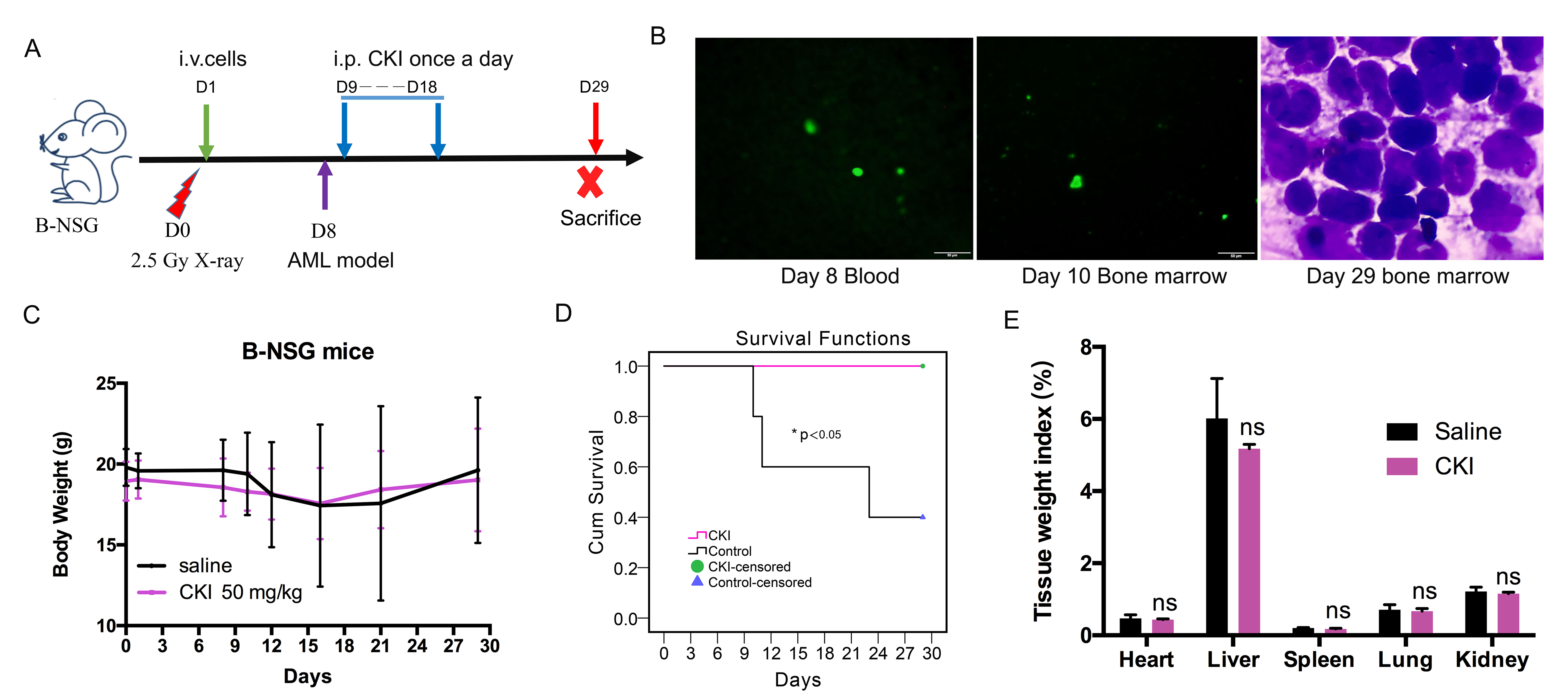

Supplement: Supplementary file 12 — Figure S8. The anti-leukaemic effects of CKI on B-NSG mice with Molm-13 GFP+ cell injections. (A) A schematic diagram of the AML animal model. (B) Analysis of the blood and bone marrow smears. At day 8, the blood was collected from the tail vein using a capillary tube at day 8 after injection of the Molm-13 GFP+ cells and blood smears were performed. At day 10, bone marrow smear analysis was performed to detect Molm-13 GFP+ cell targeting. The fluorescence intensity was observed by fluorescence microscopy. Magnification fold: ×20. At day 29, the bone marrow smears were stained and leukaemia cells were observed. Magnification fold: ×100 under an oil immersion lens. (C) The changes in body weight. (D) The survival analysis. (E) The tissue weight index. (TIF 4232 kb) [file 13046_2018_948_MOESM12_ESM.tif]
