# Supplementary material for: Compound kushen injection suppresses human acute myeloid leukaemia by regulating the Prdxs/ROS/Trx1 signalling pathway
Source: J Exp Clin Cancer Res. 2018 Nov 19;37:277. doi: 10.1186/s13046-018-0948-3 (PMC6245615; doi:10.1186/s13046-018-0948-3)
Supplement: Supplementary file 3 — Supplemental methods. (DOCX 23 kb) [file 13046_2018_948_MOESM3_ESM.docx]

**Additional file 3**

**Supplemental methods**

*T Cell culture*

T cells were separated from healthy volunteers who received medical examinations using a peripheral blood monocyte separation kit (TBD, lot: TBD2011H). T cells were cultured in RPMI 1640 media containing 10% human AB serum with 50 μM of 2-mercaptoethanol, 100 IU/ml of interleukin-2 (IL-2) and 0.1 mM MEM non-essential amino acid solution.

*Cell growth*

Trypan blue assays were used to examine cell growth. The AML cells (THP-1, U937, HL60, Molm-13 cells and human cells) were seeded into 6-well plates at 1×10^5^-10^6^ cells per well and treated with CKI for 48 h. Then, the cells were harvested and trypan blue was added at a final concentration of 0.2% to test the cell viability with a cell counter (Cellometer^®^ Mini, Nexcelom, Bioscience).

*Cell apoptosis and cell cycle*

Cell apoptosis was measured using an Annexin V-FITC/PI apoptosis kit (MultiSciences (Lianke) Biotech Co., Ltd.) according to the manufacturer’s instructions. In total, 1×106 AML cells per well were seeded into a 6-well plate and treated with CKI. After incubation for 48 h, the cells were harvested and stained with Annexin V-FITC/PI. The apoptotic cells were measured by flow cytometry (Cytomics FC 500, Beckman coulter).

To examine the changes in the mitochondrial membrane potential after CKI treatment for 24 h, U937 and THP-1 cells were stained with JC-1 dye (Beyotime, No. C2006). The change in the fluorescence intensities for JC-1 from aggregates to monomers was calculated to analyse early apoptosis by flow cytometry.

Cell cycle was examined using a kit (KeyGen Biotech, Cat. NO: KGA512) according to the manufacturer’s instructions. U937 and HL60 cells were collected and fixed with 70% ethyl alcohol at 4 ℃ for 2 h, suspended, washed with PBS, stained with propidium (PI) at 4 ℃ for 30 min avoiding light and analysed by flow cytometry.

### *Dimethylation labelling reaction and hybrid quadrupole-TOF LC-MS/MS*

In total, 200 μg peptides from each group (control and CKI treatment in U937 cells) was re-suspended in 1 ml 0.1 M NaAc (pH 5.99). The peptides were labelled by reductive methylation of the primary amino groups (N-terminal and K). Control and CKI treatment samples were distinguished as light- and intermediate-labelled by incubating with 160 µL 4% CH_2_O (light formaldehyde) and 4% CD_2_O (intermediate formaldehyde), respectively, in the presence of sodium cyanoborohydride (NaBH_3_CN) for 45 min at RT. After labelling, two differentially labelled samples were pooled at a 1:1 (w/w) ratio and desalted prior to the MS detection.

The data analysis parameters were as follows: (1) Sample type: dimethyl+0 and +4 (peptide labelled); (2) Cys. alkylation: iodoacetamide (IAA); (3) Digestion: trypsin; (4) Instrument: TripleTOF 5600; (5) Species: *Homo sapiens*; and (6) Search Effort: rapid. All peptides used for the calculation of the protein ratios were unique for a given protein or proteins within the group. The protein confidence threshold cut-off was set at greater than 0.05 (Unused ProtScore) and with at least one peptide with 95% confidence.

*Quantitative real-time PCR (qPCR)*For qPCR, the total RNA was extracted with the TRIzol method (Invitrogen) from AML cells. Then, cDNA was synthesized from 1 μg total RNA with the Reverse Transcription kit (Promega). All qPCR reactions were performed with the rTaq polymerase system (SYBR green, Sigma) for 35 cycles using a 96-well CFX Connect^TM^ fluorescence quantitative PCR instrument (Bio-rad). The PCR primer sequences were as follows: (1) human GAPDH forward (GGAGTCCACTGGCGTCTTCA) and reverse (GTCATGAGTCCTTCCACGATACC), (2) Prdx3 forward (AGTTGTCGCAGTCTCAGTGG) and reverse (AACAGCACACCGTAGTCTCG), (3) Prdx2 #1 forward (GTGTCCTTCGCCAGATCACT) and reverse (ACAAACTTCCCCATGCTCGT), and (4) Prdx2 #2 forward (GGACTCTCAGTTCACCCACC) and reverse (TTTCAGCACGCCGTAATCCT).

**Supplemental information**

**Table S1. The information of new diagnosed AML patients.**

**Table S2. The identified proteins by LC-MS/MS.**

**Table S3. The identified 54 differentially expressed proteins.**

**Figure S1. The component of CKI.**

**Figure S2. The SOD vitality after CKI treatment.**

**Figure S3. The cell growth.**

Cell growth was tested by trypan blue staining to analyse the cell count after 110 μl/ml CKI treatment for 48 h.

**Figure S4. Cell apoptosis and cell cycle for HL60 cells and THP-1 cells.**

(A) Cell apoptosis was tested by flow cytometry after HL60 cells were treated with 110 μl/ml CKI for 48 h. (B) Cell cycle was analysed by flow cytometry after HL60 cells were treated with CKI for 24 h. (C) Early apoptosis was determined by JC-1 assay after THP-1 cells were treated with 110 μl/ml CKI for 24 h.

**Figure S5. Workflow for the quantitative proteomics with dimethylation labelling after U937 cells were treated with CKI.**

**Figure S6. The proteins interacting with Prdx2 by STRING analysis.**

**Figure S7. The mRNA expression levels of Prdx2 and Prdx3 after CKI treatment.**

**Figure S8. The anti-leukaemic effects of CKI on B-NSG mice with Molm-13 GFP+ cell injections.**

(A) A schematic diagram of the AML animal model. (B) Analysis of the blood and bone marrow smears. At day 8, the blood was collected from the tail vein using a capillary tube at day 8 after injection of the Molm-13 GFP+ cells and a blood smear was performed. At day 10, bone marrow smear analysis was performed to detect Molm-13 GFP+ cell targeting. The fluorescence intensity was observed by fluorescence microscopy. Magnification fold: ×20. At day 29, the bone marrow smear was stained and leukaemia cells were observed. Magnification fold: ×100 under an oil immersion lens. (C) The changes in body weight. (D) The survival analysis. (E) The tissue weight index.
